# Breastfeeding knowledge and attitudes of health professional students: a systematic review

**DOI:** 10.1186/s13006-018-0153-1

**Published:** 2018-02-20

**Authors:** Shu-Fei Yang, Yenna Salamonson, Elaine Burns, Virginia Schmied

**Affiliations:** 1 School of Nursing and Midwifery, Western Sydney University, Penrith, NSW Australia; 2 Centre for Applied Nursing Research (CANR), Ingham Institute for Applied Medical Research, Liverpool, NSW Australia; 3 Department of Nursing, Chung Hwa University of Medical Technology, Tainan, Taiwan

**Keywords:** Breastfeeding, Breastfeeding knowledge, Breastfeeding attitudes, Nursing students, Health professional students, Literature review

## Abstract

**Background:**

Breastfeeding support from health professionals can be effective in influencing a mother’s decision to initiate and maintain breastfeeding. However, health professionals, including nursing students, do not always receive adequate breastfeeding education during their foundational education programme to effectively help mothers. In this paper, we report on a systematic review of the literature that aimed to describe nursing and other health professional students’ knowledge and attitudes towards breastfeeding, and examine educational interventions designed to increase breastfeeding knowledge and attitudes amongst health professional students.

**Methods:**

A systematic review of peer reviewed literature was performed. The search for literature was conducted utilising six electronic databases, CINAHL, MEDLINE, ProQuest, PubMed, Scopus, and Cochrane, for studies published in English from January 2000 to March 2017. Studies focused on nursing students’ or other health professional students’ knowledge, attitudes or experiences related to breastfeeding. Intervention studies to improve knowledge and attitudes, were also included. All papers were reviewed using the relevant Critical Appraisal Skills Programme (CASP) checklist.

**Results:**

Fourteen studies were included in the review. This review indicates that in some settings, health professional students demonstrated mid-range scores on breastfeeding attitudes, and their knowledge of breastfeeding was limited, particularly in relation to breastfeeding assessment and management. All of the studies that tested a specialised breastfeeding education programme, appeared to increase nursing students’ knowledge overall or aspects of their knowledge related to breastfeeding. Several factors were found to influence breastfeeding knowledge and attitudes, including timing of maternal and child health curriculum component, previous personal breastfeeding experience, gender, cultural practices and government legislation.

**Conclusions:**

Based on this review, it appears that nursing curriculum, or specialised programmes that emphasise the importance of breastfeeding initiation, can improve breastfeeding knowledge and attitudes and students’ confidence in helping and guiding breastfeeding mothers.

## Background

To achieve the health and optimal growth of infants, the World Health Organization (WHO) and the United Nations Children’s Fund (UNICEF) recommends that all infants should be exclusively breastfed for the first 6 months and continue to receive breast milk until 2 years of age to supplement other foods [1]. In addition, the policy statement of American Academy of Paediatrics cites breastfeeding as the ideal form of infant nutrition, providing health benefits for both mothers and infants [2].

There are a range of factors known to influence a mother’s decision to initiate and maintain breastfeeding including the practical, emotional support, and encouragement from health professionals [3]. A Cochrane Review reported that breastfeeding support from health professionals can be effective in extending the duration of breastfeeding [4]. It is therefore important that nursing students and other students in other health professions, acquire knowledge about breastfeeding, and develop skills to support and provide appropriate care to pregnant women, and to mothers with infants, in order to support mothers to breastfeed [5]. However, health professionals, including nursing students, do not always receive adequate breastfeeding education during their foundational education programme to effectively help mothers [6, 7]. There have been two reviews of breastfeeding educational interventions to build capacity in health professionals [8, 9]. Spiby et al. [8] identified a range of educational interventions for healthcare professionals aiming to increase knowledge and support breastfeeding, however due to methodological limitations, they were not able to support any specific approach. Watkins and Dodgson [9] found that educational interventions that mostly focused on increasing women’s knowledge about breastfeeding, and how to best support breastfeeding, may be effective in modifying maternal behaviour and healthcare providers’ understanding. To date there have been no reviews of interventions to increase the capacity of nursing or other health professional students to support breastfeeding mothers.

In this paper, we report on a systematic review of the literature that aimed to: 1. describe nursing and other health professional students’ knowledge and attitudes towards breastfeeding, and report their confidence in supporting women to breastfeed and 2. examine educational interventions designed to increase breastfeeding knowledge and attitudes amongst health professional students. The review addressed two questions: 1. What is the knowledge, attitudes and confidence of nursing students and other health professional students related to breastfeeding? and 2. Do educational interventions in addition to the standard curriculum, better prepare nursing students and other health professional students to support breastfeeding?

## Methods

### Search strategies

The search for literature was conducted utilising six electronic databases, CINAHL, MEDLINE, ProQuest, PubMed, Scopus, and Cochrane, for studies published in English from January 2000 to March 2017. As nursing curricula change over time, and to select recent publications, the year 2000 was chosen as the start date for the search. The following Medical Subject Headings (MESH) were used in combination: breastfeeding, nursing students, student nurses, medical students and health professional students. Only papers that had examined nursing students’ or other health professional students’ knowledge, attitudes or experiences related to breastfeeding were included. Intervention studies to improve knowledge and attitudes were also included.

Two frameworks were used in determining the inclusion criteria for this review. For non-intervention studies, the PEO (Person; Exposure; Outcome) framework was used: the person was defined as nursing and health professional students; the exposure was the current undergraduate educational programmes related to breastfeeding; the outcomes were defined as knowledge, attitudes or confidence towards breastfeeding. The PICO (Person; Intervention; Control; Outcome) framework was used for intervention studies [10]. Here the nursing and health professional students were the person, the intervention was the ‘add-on’ or specialised breastfeeding educational programme, the control sought to include other nursing and health professional students, and the outcome was breastfeeding knowledge, attitudes or confidence.

### Selection process

**Fig. 1:**
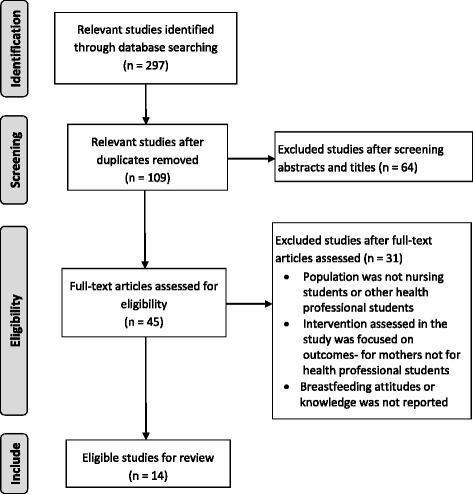
PRISMA flow diagram of study selection

A total of 297 papers were exported to the EndNote database, of these 109 were duplicates leaving 188 papers. The titles and abstracts were screened for relevance and a further 64 were removed. 45 papers were read in full and 31 were excluded because: the population was not health professional students; the intervention assessed in the study focused on outcomes for mothers and was not related to health professional student education; or breastfeeding attitudes or knowledge were not reported. One quasi-experimental study reported the findings for nursing students, but the results for the nursing students could not be distinguished from other participants and this paper was therefore excluded. One of the studies focused on midwifery students, it was determined that midwifery students spend extensive periods of time in clinical placement where they can learn about breastfeeding in contrast to nursing, and other health professional students, therefore this study was ultimately excluded. The selection process of the included papers is displayed in Fig. 1.

### Quality appraisal of studies

Fourteen papers met the inclusion criteria and were critiqued using the Critical Appraisal Skills Programme (CASP) tools to evaluate the quality of each study. Two CASP checklists were used, the qualitative research checklist consisted of 10 questions and the cohort study checklist consisted of 12 questions. Qualitative papers may achieve a total score of 10 points [11]. There were 12 questions in the cohort study checklist, with two questions scoring up to 2 points for a total of 14 points when all criteria were met [12]. Two authors read each paper and all authors discussed the scores assigned to each paper. We noted a number of study limitations including sample size and methodological issues and these are discussed in the limitations section. No studies were excluded on quality appraisal. The results of CASP scores can be found in Table 1.

**Table 1:** Summary of the characteristics of the included studies

Author(s) and country	Aim	Sample and setting	Design	Methods/instrument	Outcomes	CASP
Kakrani [20], 2015 India	To examine the knowledge levels of medical and nursing students about 10 steps of Baby-Friendly Hospital Initiative (BFHI) and to find out the gap in their knowledge about BFHI steps.	Fourth-year nursing students (*n* = 96) and third-year medical students (*n* = 102) in 1 medical college	Cross-sectional study	Questionnaire that was used to assess the knowledge gap with multiple choice questions regarding the 10 steps of successful breastfeeding	The average level of awareness among nursing (mean score: 5.84) as well as medical (mean score: 5.39) students about the ten steps.	9/14
Amin [17], 2014 Saudi Arabia	To explore the attitudes and knowledge of undergraduate female medical and education students about breastfeeding.	Medical students (*n* = 198) and students from College of Education (*n* = 323) from one university	Cross-sectional study	1. The 14 closed-ended breastfeeding knowledge questions (true-false and don’t know options or multiple choice options) 2. 17-item Iowa Infant Feeding Attitude Scale (IIFAS)	Students in the advanced years at both colleges, those who were married (22.1%) and those of rural origin (44.7%) had higher positive attitudes towards breastfeeding.	8/14
Dodgson [27], 2014 USA	To examine the beliefs and attitudes of health science university students toward formula feeding and breastfeeding.	Health science undergraduate students (*n* = 405; nursing student *n* = 84) and graduate students (*n* = 102; nursing student *n* = 52) recruited across 5 different programs at the colleges.	Cross-sectional study	Web-based validated survey instruments were used to assess the Theory of Planned Behaviour variables. These were: 1. The 19-item beliefs scale about the outcomes of breast and formula feeding 2. A 6-item breastfeeding and 6-item formula-feeding attitudes 7-point Likert scale	Significantly more positive breastfeeding attitudes and beliefs were found in graduate students (*p* = 0.0083)	9/14
Pajalic [15], 2014 Sweden	To describe nursing students’ perspectives on promoting successful breastfeeding.	Nursing students (*n* = 65)	Qualitative study (Data retrieved form of written reflections)	All the students received a paper with one open-ended question: *What do you consider success factors that promote breastfeeding in Sweden?*	Information about the benefits of breastfeeding, traditions and cultural acceptance of breastfeeding practice, and government prohibition of infant formula were important factors in promoting successful breastfeeding.	7/10
Vandewark [13], 2014 USA	To examine the relationships and change between breastfeeding knowledge and attitudes in undergraduate nursing students at the beginning and at the end of their clinical education.	Sophomore (*n* = 40) and senior (*n* = 49) nursing students from two cohorts (Second-degree and traditional 4-year students)	Mixed methods study	1. The 22-item breastfeeding knowledge instrument was adapted from Brodribb et al.’s Breastfeeding Knowledge Questionnaire (BKQ) 2. The attitude component of this survey was based on 17-item IIFAS with 3 additional questions by Ahmed and El Guindy 3. Five open-ended questions	Only knowledge scores increased with progression in their nursing studies. Attitude scores did not differ significantly between two groups. Senior students reported to be more knowledgeable about breastfeeding following their nursing education, and sophomore students believed that they would learn about breastfeeding during their course work.	10/14
Ahmed [6], 2011 USA	To assess breastfeeding knowledge among senior nursing students and to identify the types of breastfeeding knowledge among these students. To investigate the relationship between the different types of breastfeeding knowledge.	Nursing students (*n* = 115) who had completed maternal/child nursing didactic and clinical courses at two universities	Cross-sectional study	Questionnaire adapted from BKQ consisted of 24 items were classified into 3 subscales: benefits of breastfeeding, physiology of lactation, and breastfeeding management	There was a significant difference in students’ knowledge levels regarding the benefits of breastfeeding and breastfeeding management. There was also a positive relationship between students’ knowledge about physiology of lactation and breastfeeding management.	10/14
Ahmed [16], 2011 Egypt	To assess breastfeeding knowledge, attitudes and perceived adequacy of breastfeeding education of nursing students, and to investigate their self-confidence to provide breastfeeding support for mothers.	Nursing students (*n* = 92) who had completed maternal/child nursing didactic and clinical courses.	Cross-sectional study	A 24-item breastfeeding knowledge questionnaire adapted from BKQ, 17-item IIFAS, and three questions related to self-confidence and adequacy of breastfeeding education.	Low mean knowledge score of 52%. Students’ breastfeeding attitudes were unexpectedly neutral.	12/14
Brodribb [18], 2008 Australia	To describe the relationship between the cumulative length of personal breastfeeding duration and the breastfeeding knowledge, attitudes, confidence of Australian general practice (GP) registrars	Australian GP registrars (*n* = 483) in their final year of training.	Cross-sectional study	Investigator-developed Australian Breastfeeding Knowledge and Attitude Questionnaire (ABKAQ) consisting of 40-item knowledge scale and 20-item attitude scale.	The length of personal breastfeeding duration was found to influence confidence with breastfeeding. Registrars with brief personal breastfeeding duration (< 26 weeks) had lower breastfeeding attitudes, their knowledge levels were similar to doctors who had never breastfed.	9/14
Spear [19], 2006 USA	To assess basic breastfeeding knowledge and selected attitudes of junior and senior baccalaureate nursing students. To determine the need for inclusion of more in-depth information about breastfeeding in the undergraduate obstetric nursing course curriculum.	Junior (*n* = 32) and senior (*n* = 48) baccalaureate nursing students at a private university in the United States.	Cross-sectional study	1. Modified version of Smith’s (2004) 20-item breastfeeding knowledge questions 2. One open-ended attitude question about breastfeeding	Higher knowledge score was associated with positive attitudes toward breastfeeding.	9/14
Cricco-Lizza [14], 2006 USA	To explore the breastfeeding attitudes and beliefs of junior nursing students.	Nursing students (*n* = 12) newly enrolled in an urban university baccalaureate nursing program in the United States.	Qualitative study (in-depth semi structured interview)	Broad open-ended questions were used to elicit the participants’ thoughts, feelings, and experiences with breast-feeding and formula feeding.	Nursing students’ personal experiences were important in developing their breastfeeding attitudes and beliefs. Students came from a predominantly breastfeeding family had the most positive attitudes towards breastfeeding.	9/10
Davis [23], 2015 USA	To determine the effect of an evidence-based educational intervention on baccalaureate nursing students’ knowledge and attitudes in relation to breastfeeding support provided for mothers.	Baccalaureate nursing students: Intervention group (*n* = 56); Control group (*n* = 57), at a public university	Intervention:1 h lecture for all students. Experimental group -simulation role-play with a standardized patient Control group - watched a breastfeeding video	Pre/post-test ABKAQ: 36 items for knowledge and 18 items for attitude	Significant difference in pre-test and post-test scores in intervention group students’ breastfeeding knowledge and attitudes towards breastfeeding.	11/14
Cianelli [22], 2014 USA	To analyse the development of an online computer based breastfeeding training (BT) among undergraduate nursing students and the preliminary outcomes of this training.	Undergraduate nursing students (*n* = 82)	Intervention:16 h of online computer based breastfeeding training consisted of five modules, in-person (web)	Pre/post-test with no comparison group. 74 multiple choice knowledge questions, and 9 items related to confidence.	Statistically significant difference between pre and post-test knowledge assessments in all of the five modules of the breastfeeding training.	8/14
Bozzette [21], 2013 USA	To examine changes in nursing students’ knowledge after receiving content on breastfeeding and lactation in obstetrical course during their baccalaureate nursing education.	Fourth-year nursing students (*n* = 24)	Intervention:1.5 h of lecture utilizing audiovisual and written materials, in-person	Pre/post-test 20-item breastfeeding knowledge questions (true-false option) adapted from the knowledge instrument developed by Marzalik (2004)	The breastfeeding education program significantly increased students’ breastfeeding knowledge of the benefits and nutritional value and management of lactation.	9/14
Dodgson [24], 2007 Hong Kong	To determine the effectiveness of an infant feeding educational intervention on nursing students’ knowledge levels	Nursing students: Intervention group (*n* = 111, fourth-year); Control group (*n* = 162, first and second-year)	Intervention:10 h of didactic instruction, in-person; 8 weeks of perinatal clinical placement	Post-test 19-item knowledge survey (true-false and don’t know options) A 6-item breastfeeding and 6-item formula-feeding attitudes 7-point Likert scale	Control group scored significantly lower on breastfeeding knowledge than the intervention group.	8/14

## Results

### Characteristics of the included studies

Studies included in this review were conducted in seven countries: United States of America (USA), Australia, Hong Kong, Sweden, India, Egypt and Saudi Arabia. The studies included nursing students, medical students and general practice registrars. See Table 1 for summary of the characteristics of the included studies.

Study designs varied: seven were cross-sectional surveys and one was a mixed-methods study that assessed nursing students’ knowledge and or attitudes towards breastfeeding [13]. Two were qualitative studies that explored nursing students’ attitudes and beliefs about breastfeeding [14, 15] and their perspectives on promoting successful breastfeeding [15]. Three studies reported the validity and reliability of the instruments used [16–18]. Some of these studies collected data before and/or after standard theory and laboratory based learning in maternal and child health curriculum and or clinical placement [6, 13, 16, 17, 19, 20]. However these studies were not designed to evaluate the standard curriculum.

In addition, four papers reported quasi-experimental studies that tested a specific breastfeeding educational intervention designed to improve knowledge and attitudes, three were in the USA [21–23] and one in Hong Kong [24]. Two used a simple pretest and post-test method to measure nursing students’ knowledge related to breastfeeding [21, 22], and two had recruited comparison groups to compare the breastfeeding knowledge and attitudes of nursing students in both the intervention and comparison groups [23, 24]. The study by Dodgson and Tarrant [24] assessed nursing students’ breastfeeding knowledge and attitudes after they received the educational intervention, but without a baseline or pretest situation.

### Measuring breastfeeding knowledge and attitudes

Several measures including subscales of larger tools were used to measure the breastfeeding knowledge and attitudes of health professional students or other participants. These were the Australian Breastfeeding Knowledge and Attitude Questionnaire [6, 13, 16, 18, 23] and the Iowa Infant Feeding Attitude Scale [6, 13, 17, 25].

Several studies assessed nursing students’ knowledge related to breastfeeding by using modified survey tools with true/false, or don’t know, or multiple-choice questions [17, 19–22, 24]. Two studies measured the participants’ beliefs about the outcomes of, and attitudes towards, breastfeeding and formula-feeding using a modified version of the Minnesota Infant Feeding Questionnaires [26] using a 12-item scales with a 7-point response format [24, 27].

### Breastfeeding education received by students

#### Standard curriculum

Some studies described the current undergraduate curriculum indicating that breastfeeding was addressed during the maternal and child health subject. If described, these standard curriculum primarily consisted of classroom and clinical components, discussing topics such as the properties of breast milk, benefits of breastfeeding for both mother and infant, assessment parameters for effective breastfeeding, maternal support, and achievement of proper latch in a didactic instructional manner [6, 16, 19, 20]. In addition, practical experience was offered through clinical placement at a hospital based maternity unit where nursing students had opportunities to observe and interact with lactation consultants and nurses as they provided breastfeeding support for new mothers [6, 13, 16, 19].

#### Specific breastfeeding education interventions

The format and length of the specialised breastfeeding education programmes in the four studies included in this review varied. Two of the education programmes were based on the Baby Friendly Hospital Initiative (BFHI) 20-h breastfeeding curriculum [22, 24], one adapted this content in five online modules requiring 16 h to complete [22], and the other provided the theoretical content in 10 h of face-to-face instruction, and 8 weeks of clinical placement [24]. Learning content in the four studies consisted of evidence based breastfeeding information [21–24].

### Health professional students’ knowledge about breastfeeding

Overall, the cross-sectional, descriptive studies found that nursing students, medical students and general practice registrars lacked knowledge about breastfeeding even after completing their maternal and child health unit of study, particularly in relation to how best to support mothers and infants and to intervene if necessary [6, 16–20]. Two studies, one in Egypt [16] and one in the USA [6] reported that nursing students had higher knowledge about the benefits of breastfeeding for the baby and the cost benefits for families and society of breastfeeding but their knowledge of breastfeeding physiology and management was low, even following theoretical and laboratory based clinical education in their course in maternal and child nursing. Amin et al. found the breastfeeding knowledge scores were low irrespective of the educational disciplines of the students [17]. The responses of medical students and students from College of Education reflected the prevalence of many misconceptions regarding the timely initiation, duration and exclusivity of breastfeeding [17]. The study by Kakrani et al. explored the knowledge of senior medical and nursing students about the 10 steps of the BFHI in India, and found there was an average level of awareness among nursing and medical students about the ten steps [20]. They also found that female students were more aware of these BFHI steps than males after the breastfeeding education [20].

Three studies compared junior and senior nursing students on their knowledge of breastfeeding [13, 19, 24] including benefits, physiology and management. In one study both the junior and senior nursing students were aware of the benefits and physiology of breastfeeding at the two points in time, but knowledge of the management of breastfeeding was significantly higher in the group of graduating students [13]. In one qualitative study, the researcher assessed nursing students’ knowledge of breastfeeding with their written responses to one question: “What do you consider success factors that promote breastfeeding in Sweden?” [15]. Most nursing students reported that promoting breastfeeding was important for infant health, and most students demonstrated knowledge about the advantages of breastfeeding, such as “breast milk provides stronger immune protection for the child than formula milk” [15].

Of the four studies that examined a breastfeeding education programme, each reported a significant positive difference between pre and post-test breastfeeding knowledge scores [21–24]. In one study the breastfeeding educational programme was effective in increasing nursing students’ knowledge of the benefits and nutritional value of breastfeeding and management of lactation problems [21]. Moreover, the 16-h online breastfeeding training increased nursing students’ level of knowledge related to breastfeeding and the majority believed that they were fully able to perform skills to support breastfeeding [22].

### Health professional students’ attitudes towards breastfeeding

Cricco-Lizza used a qualitative approach to investigate the breastfeeding attitudes, beliefs, and personal experiences of nursing students (*n* = 12) at the beginning of their formal course work in maternal and child nursing, and the researcher suggested that nursing students’ positive attitudes towards breastfeeding were crucial for promoting breastfeeding initiation [14]. In Egypt, Ahmed and El Guindy [16] reported midrange scores on breastfeeding attitudes amongst nursing students and Vandewark [13] found similar mid-range scores for nursing students in the USA with no differences between the mean breastfeeding attitudes scores of junior and senior nursing students.

One study in the USA found that over one third of nursing students believed that women should not breastfeed in public [19] and another reported that all students held this belief [14]. Two intervention studies compared the breastfeeding attitudes of nursing students in both the intervention and comparison groups [23, 24] and found the educational intervention did not change students’ attitudes towards breastfeeding but it did alter attitudes towards formula feeding, with students being less favourable toward this practice [24]. Studies also reported a positive correlation between attitudes towards breastfeeding and breastfeeding knowledge [13, 14, 16, 19]. Ahmed and El Guindy found that despite Egyptian nursing students having low knowledge scores and not holding strongly positive attitudes towards breastfeeding, more than 70% of the students indicated they were confident or very confident about their ability to support breastfeeding [16].

### Factors influencing breastfeeding knowledge and attitudes

In these studies, several factors were found to influence the level of breastfeeding knowledge and attitudes towards breastfeeding, including stage of student enrolment (for example first year versus final year students), previous personal breastfeeding experience, gender, cultural practices and government legislation.

In the Australian study of GP registrars, Brodribb et al. found that more than 52 weeks personal breastfeeding experience was associated with higher mean breastfeeding knowledge scores, and GP registrars who had personal experience of breastfeeding were more confident in supporting women [18]. For nursing students, important factors for a positive attitude to breastfeeding included: coming from a predominantly breastfeeding family [14], or being married, or originating from a rural area [17]. The study by Pajalic showed that nursing students’ beliefs about the benefits of breastfeeding were influenced by traditions and cultural acceptance of the practice, and government restrictions on infant formula [15].

## Discussion

This review has synthesised the findings of 14 studies that assessed nursing and other health professional students’ knowledge and attitudes towards breastfeeding and included four studies that specifically tested an educational intervention to improve breastfeeding knowledge.

The findings indicate that in some settings health professional students’ knowledge of breastfeeding was limited, particularly in relation to breastfeeding assessment and management, and did not necessarily improve following the completion of a standard curriculum. Exposure to breastfeeding, either through the course, or personally, was associated with more positive attitudes towards breastfeeding amongst health professional students. Only two studies assessed health professional students’ confidence to support breastfeeding women and Egyptian nursing students appeared highly confident despite low levels of breastfeeding knowledge [16].

The four studies that examined a specialised breastfeeding educational program appeared to increase nursing students’ knowledge overall or aspects of their knowledge related to breastfeeding, as did the standard curriculum in some of the studies [13, 19, 23, 24]. In contrast, attitude towards breastfeeding did not appear to be altered by the educational interventions however one study reported that nursing students held less positive attitudes about infant formula after the intervention [24].

The second step of the Ten Steps to Successful Breastfeeding states that all healthcare staff should be trained in skills necessary to implement this policy [28, 29]. The included studies emphasised the importance of health professional students receiving education on breastfeeding and the skills to support new mothers to breastfeed. Despite this, there is variability in the quality of breastfeeding support provided by health professionals, particularly in-hospital postpartum care, and many women are dissatisfied with breastfeeding support and information they receive [3, 30]. This implies a gap, both in current undergraduate education, or ongoing education post registration, and in practice. Both nursing and medical students are educated about a wide variety of health concerns and conditions and breastfeeding education may not be prioritised [31]. If students receive no, or limited, education before their clinical placement, this may compromise the information and support women are offered. Furthermore, nursing students in the USA, Egypt, China, Taiwan, and elsewhere, provide care to women in postpartum units, under supervision [6, 16, 32] and if the supervising health professionals have not had adequate ongoing education they may not appropriately support and mentor the students.

This review highlights the need to determine how breastfeeding knowledge and skills are best facilitated in undergraduate curricula to help students relate theoretical breastfeeding knowledge to practice. Commentators emphasise the need for standardised breastfeeding education curricula to ensure that all undergraduate nursing students are taught similar core breastfeeding concepts regardless of the nursing programme attended [33].

Implementation guidelines for the Ten Steps to Successful Breastfeeding state that all healthcare staff should receive breastfeeding education including both the knowledge and skills to support women to breastfeed [28, 29]. For facility personnel whose role may involve educating, advising or assisting women in relation to breastfeeding, they must have a minimum of 20 h of breastfeeding education, consisting of at least 8 hours theoretical education and at least 3 hours relevant supervised clinical experience on breastfeeding [28, 29]. The education program may include various delivery options such as workshops, face-to-face or online education [29]. Two intervention studies were based on the BFHI 20 h module but both adapted this by either reducing the content to 16 h online [22] or reducing to 10 h with a significant clinical component of 8 weeks [24]. Both had positive effects on nursing students’ breastfeeding knowledge.

However, given the constraints of generalist nursing curricula and other courses, time does not necessarily permit a 20 h module on breastfeeding. As a consequence, curricula across the globe vary in the time devoted, and content provided. For example, in the review of educational support for health professionals, Watkins and Dodgson [9] comment that the length of breastfeeding education varied considerably from 1.5 h to 24 h of face-to-face content ranging from one to eight sessions. In this present review the specialised breastfeeding programmes for nursing students varied from as little as 2 h to 16 h of didactic lecture style as well as simulation role-play or online computer based learning modules with varying impact on nursing students’ breastfeeding knowledge and attitudes.

A range of formats and educational strategies were used in the intervention. These included didactic lecture style as well as simulation and clinical placement. Strategies such as evidence based seminar updates [33] with case studies [34] have been identified as useful approaches, in conjunction with, or perhaps replacing, didactic classroom lectures. Providing students with opportunities to practise breastfeeding management skills before actually caring for clients in a clinical setting may increase confidence [16]. Increasingly online education is used in undergraduate curriculum. Recently, researchers found that the additional online module improved undergraduate nursing students’ learning as well as their confidence in the clinical setting [35]. Researchers suggest a variety of forms of educational programmes, including workshops, seminars and more traditional teaching programmes are required [20, 33, 34].

Healthcare professionals who are experienced with breastfeeding management play a crucial role in helping nursing students practise basic breastfeeding assessment skills learnt in the classroom and laboratory [6, 32]. However, this may be problematic if the healthcare professionals lack knowledge and skill or are inappropriate in the approach they take to providing breastfeeding support. The review by Watkins and Dodgson [9] and Spiby et al. [8] indicate that not all health professionals are adequately prepared, and found that many do not feel confident and knowledgeable in managing breastfeeding problems.

### Influence of socio-cultural context on breastfeeding attitudes

It was evident in the study by Pajalic from Sweden that the nursing students overall held a positive attitude towards breastfeeding [15]. Scandinavian countries are well-known for their strong public health policies supporting breastfeeding, and their experience of high levels of breastfeeding initiation and maintenance for the first 6 months after birth [36, 37]. In contrast in the USA, breastfeeding rates and the mean breastfeeding attitude scores are lower than those of many other countries [13, 38]. Participants in one study were hesitant to continue breastfeeding because feeding the baby themselves challenged their independence, and they had concerns about intimacy with breastfeeding [14].

In the Middle Eastern countries and countries like Egypt where Islam is the dominant religion, the community and women are guided by the Qur’an which supports breastfeeding [39]. It was therefore surprising that the nursing students’ attitudes towards breastfeeding, in Egypt, were neutral with low breastfeeding knowledge scores [16]. It may be that when these Middle Eastern students were surveyed they had not completed their education and lacked the clinical experience that would potentially enhance their breastfeeding knowledge and skills [16].

### Limitations

This review may be limited by the commencement date of year 2000. Other relevant papers published before this date e.g. Freed and colleagues [40] have informed our discussion. The review is also limited by the quality of some of the included studies. Most included studies had a small response rate [13, 18, 20, 27], small sample sizes [13, 16, 19, 21, 22] and participants were not randomised in the intervention studies [21, 22, 24]. Reliability or validity of the measures used to assess nursing students’ breastfeeding knowledge and attitudes were not presented in all studies. In a number of the included papers we could not determine whether the study sample was representative of the population. Some participants completed the maternal and child health course 1 year prior to the survey and therefore capacity for recall may have affected their ability to answer the questions accurately [13, 19]. Some papers do not indicate whether or not the participants had received any breastfeeding content prior to the survey [15, 27].

### Implications for education of health professionals

These studies emphasise that healthcare professionals, including nurses and doctors should participate in ongoing breastfeeding education [6, 40]. Particularly important is the need to challenge nurses’ attitudes and cultural norms related to breastfeeding, in addition to more traditional items such as the treatment methods for mastitis, to fully prepare nursing students to provide care for new mothers [19, 41].

It is interesting that no recent studies of nursing students’ breastfeeding knowledge and attitudes were conducted in the United Kingdom (UK) and Australia. This may be because midwives primarily provide breastfeeding support to women. There have been recent studies of both registered midwives’ breastfeeding knowledge and attitudes in Australia [42] and student midwives in UK [43]. It is however important that nursing and other health professional students also have the basic knowledge and skills to support breastfeeding women when they are on a postpartum unit, and that they have appropriate supervision and support from qualified health professionals [9]. It is important that nursing students in countries like UK and Australia, also receive some education related to breastfeeding because they may encounter breastfeeding women in general practices [44], paediatric wards and also in emergency departments [45].

While the findings of this review highlight the need for improvements in breastfeeding education in the baccalaureate nursing curriculum, this review provides little guidance as to what content is needed, how long the theoretical and clinical experiences should be and what is the best mode of delivery to increase knowledge and skill. The question remains whether nursing and other health professional students require 20 h of education at undergraduate level including how to manage breastfeeding problems, in order to provide the best support [46].

## Conclusion

It is essential that health professional students have a positive attitude towards breastfeeding, and are able to provide breastfeeding women with the basic information they require. From the studies reviewed, baccalaureate students are considered novices who lack basic breastfeeding knowledge. Not all health professionals are adequately prepared and feel confident and knowledgeable in managing breastfeeding problems. In conclusion, it appears that nursing students can benefit from targeted programes to increase breastfeeding knowledge and attitudes, and their confidence in helping and guiding breastfeeding mothers. To ensure that future health professionals are well prepared to support breastfeeding. It is important that the curriculum is evidence based and culturally appropriate.

## Abbreviations

CASP: Critical Appraisal Skills Programme
PRISMA: Preferred Reporting Items for Systematic Reviews and Meta-Analyses
UNICEF: United Nations Children’s Fund
WHO: World Health Organization
